# Machine learning-driven sedation-analgesia optimization in mechanically ventilated sepsis patients: a retrospective MIMIC-IV analysis

**DOI:** 10.3389/fphar.2026.1673704

**Published:** 2026-01-21

**Authors:** Qinxue Hu, Tao Xu, Xiaolan Gao, Xianying Lei, Lirong Hu

**Affiliations:** Department of Critical Care Medicine, The Affiliated Hospital, Southwest Medical University, Luzhou, China

**Keywords:** lightgbm, mechanical ventilation, mechine learning, sedation-analgesia optimization, sepsis, SHAP analysis

## Abstract

**Background:**

In the intensive care unit (ICU), septic patients frequently require endotracheal intubation followed by invasive mechanical ventilation. Nonetheless, the optimal sedation-analgesia regimen for these critically ill patients remains undetermined.

**Methods:**

This retrospective observational study analyzed data from the Medical Information Mart for Intensive Care IV (MIMIC-IV version 3.0) database to examine septic patients who underwent endotracheal intubation and subsequent invasive mechanical ventilation in the intensive care unit. Initially, Kaplan–Meier survival analysis and Cox proportional hazards models were employed to evaluate the prognostic impact of different sedation-analgesia regimens. Subsequently, the least absolute shrinkage and selection operator (LASSO) regression was utilized to identify key prognostic factors. Multiple machine learning algorithms were then implemented to develop predictive models, and the SHapley Additive exPlanations (SHAP) method was used to interpret the model outputs and determine the most influential predictors.

**Results:**

Following the initial screening process, seven distinct sedation-analgesia regimens with sample sizes greater than 100 were incorporated into the final analysis. Utilizing Kaplan–Meier estimates and Cox regression models, the combination of fentanyl and midazolam was identified as the most advantageous regimen. This association remained statistically significant after adjusting for confounding variables, demonstrating a reduction in the length of stay in the intensive care unit (length of stay in ICU, HR [95% CI]: 0.66 [0.52–0.85]) and a decrease in ICU mortality (OR [95% CI]: 0.62 [0.46–0.85]). Subsequently, LASSO regression analysis identified seven key prognostic factors associated with outcomes in this patient subgroup. Among the machine learning models developed for outcome prediction, the LightGBM model exhibited superior performance (AUC = 0.838). SHAP analysis indicated that the top three predictors of 28-day mortality were the Acute Physiology Score III (APS III), patient age, and the presence of acute renal failure.

**Conclusion:**

The concurrent administration of fentanyl and midazolam was associated with lower ICU mortality and shorter length of ICU stay among septic patients necessitating endotracheal intubation and invasive mechanical ventilation, suggesting potential clinical benefit. Furthermore, the LightGBM algorithm exhibited superior predictive accuracy for ICU mortality within this cohort, suggesting its potential utility as a tool for supporting data-driven clinical decision-making.

## Introduction

Sepsis continues to pose a significant global health challenge, with an estimated annual incidence of 50 million cases (Worldsepsisday, 2025) and a 90-day mortality rate of 35.5% (Xie et al., 2020). Among patients admitted to intensive care units, those requiring endotracheal intubation and mechanical ventilation are particularly vulnerable, facing poor prognoses due to the complex interplay of systemic inflammation, organ dysfunction, and iatrogenic complications (Hill et al., 2017; Kim et al., 2024). Current epidemiological data underscore the urgent need to enhance clinical management strategies for this at-risk population, especially concerning life-support interventions such as mechanical ventilation.

Sedative and analgesic regimens during mechanical ventilation have a profound impact on patient outcomes, influencing the incidence of delirium (Latronico et al., 2024) ventilator-associated complications (Weinberger et al., 2021), and long-term survival (Shehabi et al., 2018). However, the variability in sedation protocols—ranging from deep to light sedation or targeted analgesia—has resulted in inconsistent clinical outcomes (Aitken et al., 2021; Stephens et al., 2018). For septic patients, whose physiological reserves are already compromised by hypermetabolic states and immune dysregulation (Mira et al., 2017; Wasyluk and Zwolak, 2021), individualized sedation-analgesia strategies may help mitigate secondary organ injury and improve recovery trajectories (Shehabi et al., 2021). Nevertheless, the current body of evidence regarding optimal pharmacological strategies for sepsis-specific populations remains sparse, highlighting the urgent need to identify regimen-specific prognostic indicators.

Recent advancements in machine learning (ML) present significant potential for enhancing clinical prognostication, especially in critical care environments characterized by high-dimensional datasets (Pirracchio et al., 2019; Venturini et al., 2024; Wu et al., 2023). Techniques such as LASSO regression are particularly effective in feature selection, as they penalize non-informative variables and distill complex datasets into actionable predictors. When integrated with predictive modeling frameworks, these methods facilitate robust risk stratification and outcome prediction for critically ill patients (Wang et al., 2025; Fathy et al., 2025). This approach is particularly relevant to sepsis, where the multivariable interactions between treatment modalities and host responses complicate traditional statistical analyses.

Based on these principles, we propose that machine learning analysis of sedation-analgesia protocols can determine the best therapeutic profiles for septic patients on mechanical ventilation, while also allowing for prognostic predictions. Utilizing the MIMIC-IV database, this research employs LASSO regression to pinpoint crucial factors affecting ICU mortality and complications, followed by the development of predictive models using ensemble learning techniques. By integrating pharmacological, physiological, and outcome data, our goal is to create a framework for tailored sedation-analgesia strategies and improve prognostic precision in sepsis treatment. This combined method could eventually guide clinical practices and optimize resource distribution in ICU.

## Methods

### Study population and design

This retrospective cohort study utilized clinical records from the MIMIC-IV database (version 3.0), an open-access repository of critical care data that includes comprehensive profiles of ICU patients from Beth Israel Deaconess Medical Center, covering the period from 2008 to 2019. The dataset comprises demographic information, infection sites, comorbidities, perioperative records, therapeutic interventions (such as pharmacotherapy and fluid management), laboratory results, severity scoring metrics, and longitudinal survival data. A certified researcher QX Hu (Record ID: 69413356) conducted data curation in accordance with institutional review board protocols, which require complete de-identification through cryptographic encoding of patient information. Established through a collaborative effort between the Massachusetts Institute of Technology and the host medical center, this resource operates under waived ethical approval requirements due to its pseudonymization processes, which effectively obscure protected health information during secondary analysis. We enrolled adult sepsis patients meeting the Sepsis-3.0 criteria who underwent endotracheal intubation and received invasive mechanical ventilation for >24 h after ICU admission. Patients without endotracheal intubation, invasive mechanical ventilation, sedation, or analgesia therapy were excluded.

### Data extraction

The extracted dataset included the admission number and comprehensive patient information encompassing basic demographics (age, sex, body mass index [BMI], and marital status), type of ICU admission, as well as hospitalization and mortality outcomes. These outcomes included length of stay (LOS) in the ICU and hospital, ICU mortality, in-hospital mortality, and 1-year mortality. Vital signs recorded at the time of ICU admission included systolic blood pressure (SBP), diastolic blood pressure (DBP), respiratory rate, oxygen saturation (SpO_2_), and body temperature. Additional clinical variables included duration of mechanical ventilation and the presence of shock, defined as SBP <90 mmHg. The dataset also captured the site of inflammatory involvement, multiple clinical severity scores (Systemic Inflammatory Response Syndrome [SIRS], Sequential Organ Failure Assessment [SOFA], Acute Physiology Score III [APS III], Simplified Acute Physiology Score II [SAPS II], Oxford Acute Severity of Illness Score [OASIS], and Glasgow Coma Scale [GCS]), and a wide range of comorbidities. These comorbidities included hypertension, type 1 and type 2 diabetes mellitus, heart failure, myocardial infarction, malignant neoplasms, chronic kidney disease, acute renal failure (ARF), cirrhosis, hepatitis, tuberculosis, pneumonia, stroke, hyperlipidemia, and chronic obstructive pulmonary disease (COPD). Laboratory parameters consisted of white blood cell count (WBC), red blood cell count (RBC), platelet count (PLT), hemoglobin (HGB), albumin, serum sodium, potassium, calcium, glucose, thrombin time (TT), prothrombin time (PT), activated partial thromboplastin time (APTT), international normalized ratio (INR), D-dimer, alanine aminotransferase (ALT), aspartate aminotransferase (AST), and N-terminal pro-brain natriuretic peptide (NT-proBNP). Pharmacological treatments administered during ICU stay included antihypertensive agents, glucocorticoids, nephrotoxic drugs, and immunosuppressants. The primary outcomes assessed were hospitalization and mortality metrics, specifically the LOS in the ICU and hospital, ICU mortality, and 1-year all-cause mortality.

Sedative regimens administered during mechanical ventilation in the ICU were categorized based on the specific agents used, either as monotherapy or in combination. The agents included propofol, etomidate, fentanyl, dexmedetomidine, midazolam, hydromorphone, and morphine, as well as commonly observed combinations such as propofol with fentanyl, hydromorphone, or morphine; etomidate with fentanyl, hydromorphone, or morphine; dexmedetomidine in combination with other sedatives or analgesics; fentanyl combined with midazolam; fentanyl, midazolam, and hydromorphone; fentanyl, midazolam, and morphine; and other regimens not falling into the aforementioned categories (Supplementary Table S1). To reduce the potential impact of small sample sizes on the reliability of statistical analyses, sedative regimen groups with fewer than 100 patients were excluded from further analysis.

### LASSO regression analysis

We employed the least absolute shrinkage and selection operator (LASSO), a widely utilized regression technique that applies ℓ1 penalties to produce sparse solutions. The “glmnet” package in R was used to conduct the LASSO regression, enabling us to filter and identify the most significant predictors influencing the survival outcomes of sepsis patients. The included factors including age, sex, hypertension, type 2 diabetes, hyperlipidemia, stroke, type 1 diabetes, chronic obstructive pulmonary disease, heart failure, myocardial infarction, malignant tumor, chronic kidney disease, ARF, cirrhosis, hepatitis, tuberculosis, pneumonia, shock, ventilation duration (hours), binary body mass index, SOFA score, APS III score, SIRS score, SAPS II score, OASIS score, GCS level, anti-hypertensive drugs, glucocorticoid, nephrotoxic drug, immunosuppressant, white blood cell count, red blood cell count, platelet count, hemoglobin, albumin, serum sodium, serum potassium, total calcium, blood glucose, partial thromboplastin time, international normalized ratio, alanine aminotransferase, aspartate aminotransferase, and NT-proBNP.

### Statistical analysis

Descriptive statistics were used to summarize the distribution of quantitative variables. Parametric data were expressed as mean ± standard deviation (SD), while non-parametric data were reported as median with interquartile range (IQR). Categorical variables were presented as frequencies and percentages. For longitudinal survival analysis, Kaplan–Meier estimates and the log-rank test were employed to compare time-to-death distributions among different sedative and analgesic regimens, specifically evaluating 28-day ICU mortality, 28-day in-hospital mortality, and 1-year all-cause mortality. To assess the independent associations between sedation-analgesia regimens and mortality outcomes at 28 and 365 days, multivariable Cox proportional hazards models were constructed. Hazard ratios (HRs) with corresponding confidence intervals were calculated, adjusting for potential confounding covariates. For ICU and hospital length of stay (LOS), discharge is subject to the competing risk of death; therefore, LOS was analyzed as time to discharge alive using the Fine–Gray subdistribution hazards model, treating death prior to discharge as the competing event. Subdistribution hazard ratios (sHRs) with 95% confidence intervals were reported, where sHR >1 indicates a higher cumulative incidence of discharge over time. Following the identification of key factors associated with 28-day mortality in ICU through LASSO regression, subgroup analyses were conducted based on these variables. All subgroup analyses were adjusted for potential confounding covariates to ensure robust and unbiased estimates of association.

To construct a predictive model for 28-day ICU mortality among sepsis patients who underwent mechanical ventilation, the dataset was initially partitioned into a training set and a validation set in an 8:2 ratio. Feature selection and redundancy reduction were subsequently conducted on the training set using Lasso regression. Five machine learning models—Extreme Gradient Boosting (XGBoost), Decision Tree (DT), Light Gradient Boosting Machine (LightGBM), Logistic Regression, and Random Forest (RF)—were developed to construct predictive models for patient outcomes. The model’s performance was assessed through the maximum area under the receiver operating characteristic (ROC) curve (AUC), alongside metrics such as sensitivity, specificity, recall, F1 score, and accuracy for the validation set. A higher AUC value indicates superior discriminative capability of the model. Sensitivity and specificity measure the model’s proficiency in accurately identifying positive and negative samples, respectively. Additionally, the SHAP (SHapley Additive exPlanations) method was employed to generate bar graphs illustrating the contribution of each feature to the predicted outcome. SHAP was further utilized to evaluate the impact of specific features on individual samples, thereby enhancing the understanding of the model’s decision-making process. All statistical analyses were performed using R software (version 4.3.1) with bilateral *P*-value <0.05 considered statistically significant.

## Results

### Baseline characteristics

**FIGURE 1:**
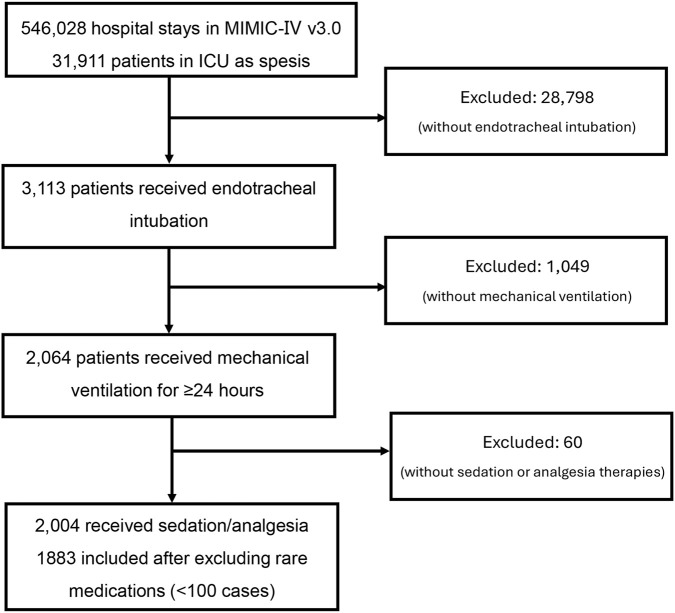
Flow chart for the patients eligible for analysis.

This retrospective study leveraged the MIMIC-IV v3.0 database to examine the effects of various sedation and analgesia regimens on the outcomes of mechanically ventilated patients with sepsis. From a total of 546,028 hospitalization records, 31,911 sepsis patients were identified, of whom 3,113 underwent endotracheal intubation, thereby excluding 28,798 non-intubated cases. Among the intubated patients, 2,064 received mechanical ventilation for a duration of 24 h or more. Following the exclusion of 60 patients who did not receive sedation or analgesia, 2,004 patients were included in the study. To improve estimation stability and avoid sparse-data problems (e.g., separation and highly uncertain effect estimates) in multivariable models, regimens with fewer than 100 patients were excluded from the primary comparative analyses (Figure 1). Consequently, six groups, encompassing a total of 1,883 patients, were selected for further analysis: 490 patients (26.2%) were administered propofol combined with fentanyl, hydromorphone, or morphine; 158 patients (8.4%) received etomidate in conjunction with fentanyl, hydromorphone, or morphine; 269 patients (14.4%) were treated with dexmedetomidine alongside other drugs; 416 patients (22.2%) received a combination of fentanyl and midazolam; 123 patients (6.6%) were given fentanyl, midazolam, and hydromorphone; and 236 patients (12.6%) received fentanyl, midazolam, and morphine. An additional 191 patients (10.2%) were treated with other protocols. The results indicated significant differences (*P* < 0.05) among groups concerning demographics, hospitalization outcomes, sites of inflammatory involvement, clinical severity scores, laboratory parameters, and interventions, including antihypertensive medications and glucocorticoid use. These findings imply that sedation and analgesia protocols may impact outcomes through multifactorial pathways, highlighting the need for further validation of data accuracy and examination of potential confounding variables. The baseline characteristics of the two cohorts are detailed in Table 1 and Supplementary Table S1, contingent upon whether the number of included cohorts exceeded 100 (Table 1; Supplementary Table S1).

**TABLE 1:** Basic information of eligible patients.

Variables	Total (n = 1883)	Propofol plus fentanyl/hydromorph/morphine (n = 490)	Etomidate plus fentanyl/hydromorph/morphine (n = 158)	Dexmedetomidine plus other drugs (n = 269)	Fentanyl plus midazolam (n = 416)	Fentanyl plus midazolam plus hydromorph (n = 123)	Fentanyl plus midazolam plus morphine (n = 236)	Others (n = 191)	*P*
Demographics									
Age, year	64.43 ± 16.78	63.58 ± 17.07	66.27 ± 17.39	60.05 ± 16.83	65.59 ± 16.69	63.05 ± 15.78	69.59 ± 14.02	63.20 ± 17.51	<0.001
Gender, n (%)	​	​	​	​	​	​	​	​	0.293
Female	850 (45.14)	67 (42.41)	112 (41.64)	202 (48.56)	58 (47.15)	114 (48.31)	91 (47.64)	206 (42.04)	​
Male	1033 (54.86)	91 (57.59)	157 (58.36)	214 (51.44)	65 (52.85)	122 (51.69)	100 (52.36)	284 (57.96)	​
Marital status, n (%)	​	​	​	​	​	​	​	​	0.002
Divorced	115 (6.11)	3 (1.90)	19 (7.06)	20 (4.81)	7 (5.69)	16 (6.78)	14 (7.33)	36 (7.35)	​
Married	813 (43.18)	66 (41.77)	94 (34.94)	185 (44.47)	54 (43.90)	117 (49.58)	74 (38.74)	223 (45.51)	​
Single	587 (31.17)	55 (34.81)	107 (39.78)	131 (31.49)	38 (30.89)	59 (25.00)	60 (31.41)	137 (27.96)	​
Widowed	216 (11.47)	20 (12.66)	18 (6.69)	59 (14.18)	13 (10.57)	31 (13.14)	24 (12.57)	51 (10.41)	​
Missing data	152 (8.07)	14 (8.86)	31 (11.52)	21 (5.05)	11 (8.94)	13 (5.51)	19 (9.95)	43 (8.78)	​
BMI	29.13 ± 8.10	28.85 ± 7.52	27.95 ± 7.20	30.66 ± 9.25	29.22 ± 8.62	30.64 ± 8.71	28.13 ± 7.79	28.72 ± 6.84	0.001
BMI binary, n (%)	​	​	​	​	​	​	​	​	0.058
<18.5 or ≥25	545 (29.11)	47 (30.13)	69 (25.65)	117 (28.47)	24 (19.67)	83 (35.47)	55 (28.80)	150 (30.67)	​
18.5–25	1327 (70.89)	109 (69.87)	200 (74.35)	294 (71.53)	98 (80.33)	151 (64.53)	136 (71.20)	339 (69.33)	​
ICU type	​	​	​	​	​	​	​	​	<0.001
CCU	147 (7.81)	10 (6.33)	16 (5.95)	57 (13.70)	8 (6.50)	30 (12.71)	7 (3.66)	19 (3.88)	​
CVICU	145 (7.70)	22 (13.92)	48 (17.84)	7 (1.68)	8 (6.50)	12 (5.08)	5 (2.62)	43 (8.78)	​
MICU	607 (32.24)	43 (27.22)	78 (29.00)	175 (42.07)	33 (26.83)	83 (35.17)	49 (25.65)	146 (29.80)	​
MICU/SICU	371 (19.70)	24 (15.19)	36 (13.38)	120 (28.85)	19 (15.45)	85 (36.02)	22 (11.52)	65 (13.27)	​
SICU	339 (18.00)	25 (15.82)	48 (17.84)	39 (9.38)	23 (18.70)	20 (8.47)	58 (30.37)	126 (25.71)	​
TSICU	274 (14.55)	34 (21.52)	43 (15.99)	18 (4.33)	32 (26.02)	6 (2.54)	50 (26.18)	91 (18.57)	​
Hospitalization and mortality outcomes									
LOS in ICU, days	10.96 ± 9.12	11.28 ± 8.26	13.19 ± 12.18	12.20 ± 8.93	8.72 ± 7.25	13.30 ± 11.66	10.44 ± 8.50	10.51 ± 10.01	<0.001
LOS in hospital, days	18.34 ± 14.33	19.47 ± 13.38	22.41 ± 18.59	19.40 ± 12.80	14.25 ± 10.99	21.88 ± 13.99	16.45 ± 16.20	19.58 ± 16.51	<0.001
ICU mortality, n (%)	423 (22.46)	38 (24.05)	29 (10.78)	83 (19.95)	15 (12.20)	124 (52.54)	45 (23.56)	89 (18.16)	<0.001
Hospital mortality, n (%)	542 (28.78)	53 (33.54)	35 (13.01)	91 (21.88)	20 (16.26)	151 (63.98)	67 (35.08)	125 (25.51)	<0.001
1-year mortality, n (%)	835 (44.34)	81 (51.27)	60 (22.30)	173 (41.59)	40 (32.52)	185 (78.39)	96 (50.26)	200 (40.82)	<0.001
Vital sign									
Heart rate, bpm	95.56 ± 22.09	94.26 ± 21.56	97.13 ± 21.63	96.25 ± 22.42	94.70 ± 22.73	97.76 ± 20.94	96.36 ± 21.85	96.14 ± 22.93	0.536
SBP, mmHg	122.00 ± 26.78	123.73 ± 26.62	120.94 ± 25.91	122.87 ± 26.36	120.02 ± 25.19	121.80 ± 28.42	119.50 ± 27.71	124.74 ± 29.34	0.184
DBP, mmHg	67.27 ± 20.39	67.28 ± 20.10	66.96 ± 19.18	69.54 ± 21.12	66.10 ± 18.51	67.36 ± 23.30	66.02 ± 21.77	68.38 ± 21.17	0.400
Respiration rate, bpm	21.04 ± 7.05	21.22 ± 7.37	20.54 ± 7.06	20.53 ± 7.31	21.56 ± 6.92	21.20 ± 6.84	21.16 ± 7.10	20.26 ± 6.07	0.295
SPO_2_, %	96.22 ± 5.33	96.22 ± 4.97	96.14 ± 4.92	96.39 ± 7.17	95.83 ± 5.50	96.10 ± 4.56	96.13 ± 4.84	97.08 ± 3.95	0.265
Temperature, °C	36.50 ± 3.45	36.80 ± 2.45	36.41 ± 3.04	36.79 ± 2.45	36.15 ± 4.54	36.23 ± 4.39	36.13 ± 4.70	36.83 ± 0.96	0.018
Ventilation hour	180.65 ± 189.26	176.27 ± 159.97	222.66 ± 264.86	195.35 ± 178.93	148.06 ± 152.42	224.57 ± 248.95	187.47 ± 182.60	170.72 ± 220.15	<0.001
Shock, n (%)	​	​	​	​	​	​	​	​	0.581
No	1682 (90.09)	142 (89.87)	242 (90.30)	371 (89.83)	113 (93.39)	203 (87.50)	168 (88.42)	443 (91.34)	​
Yes	185 (9.91)	16 (10.13)	26 (9.70)	42 (10.17)	8 (6.61)	29 (12.50)	22 (11.58)	42 (8.66)	​
Score of inflammatory involvement									
Respiration	0.92 ± 1.18	0.98 ± 1.16	0.84 ± 1.22	1.23 ± 1.25	0.85 ± 1.17	0.79 ± 1.11	0.76 ± 1.12	0.81 ± 1.14	<0.001
Coagulation	0.46 ± 0.87	0.49 ± 0.88	0.56 ± 0.96	0.39 ± 0.82	0.39 ± 0.80	0.37 ± 0.78	0.58 ± 0.98	0.52 ± 0.82	0.028
Liver	0.34 ± 0.88	0.28 ± 0.79	0.40 ± 0.98	0.26 ± 0.75	0.33 ± 0.88	0.29 ± 0.80	0.45 ± 1.02	0.47 ± 0.98	0.034
Cardiovascular	1.11 ± 1.27	1.08 ± 1.24	1.04 ± 1.16	1.11 ± 1.27	1.22 ± 1.34	1.00 ± 1.19	1.29 ± 1.38	0.88 ± 1.16	0.017
CNS	0.48 ± 0.96	0.48 ± 0.93	0.48 ± 0.91	0.49 ± 1.03	0.45 ± 0.97	0.45 ± 0.85	0.48 ± 0.95	0.54 ± 1.02	0.969
Renal	0.60 ± 1.03	0.50 ± 0.94	0.59 ± 0.98	0.49 ± 0.97	0.73 ± 1.14	0.67 ± 1.06	0.69 ± 1.05	0.62 ± 1.05	0.011
Clinical severity scores									
SIRS score	3.07 ± 0.85	2.94 ± 0.87	2.97 ± 0.84	3.05 ± 0.86	3.16 ± 0.88	3.15 ± 0.80	3.24 ± 0.77	3.08 ± 0.80	<0.001
SOFA score	7.09 ± 4.00	6.64 ± 4.07	6.73 ± 3.31	6.73 ± 3.91	7.50 ± 3.94	7.07 ± 4.25	8.41 ± 4.07	6.52 ± 3.97	<0.001
APS III	60.39 ± 24.50	56.37 ± 23.44	59.22 ± 21.50	56.59 ± 24.01	64.48 ± 24.77	62.29 ± 25.17	68.23 ± 24.07	57.22 ± 26.16	<0.001
SAPS II	45.23 ± 15.65	43.33 ± 14.82	43.59 ± 14.78	42.55 ± 14.80	46.89 ± 16.00	45.10 ± 15.04	51.63 ± 15.74	43.77 ± 16.73	<0.001
OASIS	38.77 ± 8.42	38.10 ± 8.40	37.98 ± 8.87	37.67 ± 8.10	39.83 ± 8.14	38.72 ± 8.16	41.27 ± 8.30	37.27 ± 8.62	<0.001
GCS	12.87 ± 3.49	12.94 ± 3.45	13.27 ± 2.99	12.91 ± 3.54	12.92 ± 3.46	13.00 ± 3.36	12.44 ± 3.91	12.60 ± 3.51	0.310
Laboratory parameters									
WBC, K/μL	13.26 ± 8.89	13.47 ± 8.88	13.14 ± 7.45	13.48 ± 8.78	12.51 ± 7.73	14.00 ± 8.83	13.41 ± 8.93	13.50 ± 11.98	0.612
RBC, m/μL	3.58 ± 0.80	3.71 ± 0.84	3.42 ± 0.78	3.63 ± 0.80	3.55 ± 0.76	3.54 ± 0.83	3.46 ± 0.78	3.54 ± 0.77	<0.001
Plt, K/μL	206.67 ± 116.45	203.16 ± 114.64	203.33 ± 126.34	208.20 ± 110.16	214.55 ± 110.75	220.80 ± 123.96	205.81 ± 131.39	191.14 ± 107.85	0.257
HGB, mg/dL	10.77 ± 2.32	11.12 ± 2.47	10.32 ± 2.35	10.96 ± 2.34	10.71 ± 2.11	10.50 ± 2.47	10.37 ± 2.22	10.75 ± 2.21	<0.001
Albumin, g/dL	2.23 ± 1.32	2.03 ± 1.43	2.46 ± 1.18	2.35 ± 1.31	2.18 ± 1.33	2.29 ± 1.11	2.34 ± 1.14	2.30 ± 1.39	0.001
Sodium, mEq/L	138.37 ± 5.83	138.29 ± 5.45	137.34 ± 5.36	138.77 ± 5.30	138.84 ± 6.54	137.84 ± 5.44	137.76 ± 6.53	138.92 ± 5.35	0.026
Potassium, mEq/L	4.19 ± 0.81	4.13 ± 0.79	4.21 ± 0.82	4.23 ± 0.83	4.21 ± 0.89	4.20 ± 0.77	4.22 ± 0.78	4.18 ± 0.71	0.679
Calcium, mEq/L	8.06 ± 0.97	8.15 ± 0.94	8.06 ± 0.96	8.10 ± 0.99	7.94 ± 0.96	7.90 ± 1.00	8.01 ± 1.00	8.24 ± 0.99	0.002
Glucose, mg/dL	154.85 ± 82.08	152.36 ± 70.00	144.92 ± 58.15	146.08 ± 61.36	164.67 ± 110.14	158.02 ± 101.31	163.64 ± 85.60	147.49 ± 57.45	0.012
PT, sec	16.92 ± 11.35	15.97 ± 11.12	17.15 ± 10.68	16.23 ± 11.18	17.96 ± 13.01	16.57 ± 13.46	18.50 ± 10.36	16.10 ± 7.68	0.033
APTT, sec	38.69 ± 25.19	38.14 ± 24.92	37.71 ± 20.31	38.04 ± 24.40	38.95 ± 25.93	36.61 ± 25.00	41.88 ± 26.64	38.64 ± 27.20	0.504
INR	1.54 ± 1.09	1.45 ± 0.89	1.60 ± 1.35	1.47 ± 1.04	1.66 ± 1.40	1.38 ± 0.62	1.72 ± 1.09	1.46 ± 0.78	0.004
D-dimer	44.13 ± 484.27	0.00 ± 0.00	10.54 ± 132.46	0.00 ± 0.00	83.97 ± 748.10	27.90 ± 309.45	167.11 ± 864.35	18.96 ± 262.08	<0.001
ALT, IU/L	127.88 ± 584.25	99.26 ± 397.51	94.22 ± 295.43	150.91 ± 773.55	177.30 ± 761.98	84.64 ± 326.73	131.87 ± 657.01	112.01 ± 420.72	0.432
AST, IU/L	207.24 ± 946.64	190.23 ± 1009.91	181.46 ± 769.20	222.17 ± 961.11	277.25 ± 1233.28	138.51 ± 574.73	159.07 ± 512.08	202.48 ± 763.34	0.693
NTproBNP	1010.62 ± 5299.82	793.97 ± 4193.51	903.67 ± 4284.23	792.24 ± 4780.75	1183.40 ± 5874.12	875.43 ± 4750.39	1444.00 ± 6488.21	1137.75 ± 6565.55	0.738
ICU medications									
Antihypertensive	​	​	​	​	​	​	​	​	<0.001
No	351 (18.64)	17 (10.76)	31 (11.52)	99 (23.80)	21 (17.07)	58 (24.58)	41 (21.47)	84 (17.14)	​
Yes	1532 (81.36)	141 (89.24)	238 (88.48)	317 (76.20)	102 (82.93)	178 (75.42)	150 (78.53)	406 (82.86)	​
Glucocorticoids	​	​	​	​	​	​	​	​	0.017
No	1356 (72.01)	123 (77.85)	209 (77.70)	277 (66.59)	95 (77.24)	165 (69.92)	135 (70.68)	352 (71.84)	​
Yes	527 (27.99)	35 (22.15)	60 (22.30)	139 (33.41)	28 (22.76)	71 (30.08)	56 (29.32)	138 (28.16)	​
Nephrotoxic	​	​	​	​	​	​	​	​	0.007
No	271 (14.39)	14 (8.86)	32 (11.90)	64 (15.38)	8 (6.50)	34 (14.41)	37 (19.37)	82 (16.73)	​
Yes	1612 (85.61)	144 (91.14)	237 (88.10)	352 (84.62)	115 (93.50)	202 (85.59)	154 (80.63)	408 (83.27)	​
Immunosuppressant	​	​	​	​	​	​	​	​	0.011
No	1810 (96.12)	152 (96.20)	267 (99.26)	394 (94.71)	119 (96.75)	230 (97.46)	177 (92.67)	471 (96.12)	​
Yes	73 (3.88)	6 (3.80)	2 (0.74)	22 (5.29)	4 (3.25)	6 (2.54)	14 (7.33)	19 (3.88)	​
Comorbidity, n (%)	​	​	​	​	​	​	​	​	<0.001
No	103 (5.47)	6 (3.80)	21 (7.81)	16 (3.85)	10 (8.13)	4 (1.69)	23 (12.04)	23 (4.69)	​
Yes	1780 (94.53)	152 (96.20)	248 (92.19)	400 (96.15)	113 (91.87)	232 (98.31)	168 (87.96)	467 (95.31)	​

Categorical variables are expressed as n (%) and continuous variables are expressed as mean ± SD. BMI, body mass index; ICU, intensive care unit; CCU, coronary care unit; CVICU, cardiovascular intensive care unit; MICU, medical intensive care unit; SICU, surgical intensive care unit; TSICU, trauma surgical intensive care unit; LOS, length of stay; SBP, systolic blood pressure; DBP, diastolic blood pressure; SPO2, peripheral capillary oxygen saturation; CNS, central nervous system; SIRS, systemic inflammatory response syndrome; SOFA, sequential organ failure assessment; APS III, acute physiology score III; SAPS II, simplified acute physiology score II; OASIS, oxford acute severity of illness score; GCS, glasgow coma scale; WBC, white blood cell; RBC, red blood cell; Plt, platelet count; HGB, hemoglobin; ALT, alanine aminotransferase; AST, aspartate aminotransferase; NTproBNP, N-terminal pro b-type natriuretic peptide; PT, prothrombin time; APTT, activated partial thromboplastin time; INR, international normalized ratio.

### Relationship between fentanyl plus midazolam and survival outcomes

**FIGURE 2:**
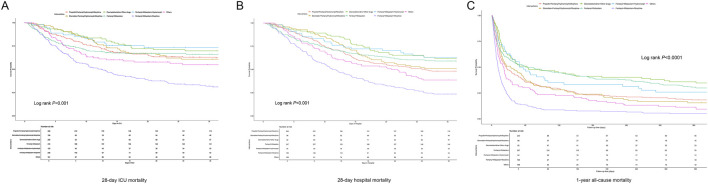
Prognostic Impact of Fentanyl Plus Midazolam in Mechanically Ventilated Septic ICU Patients Kaplan–Meier survival curves were utilized to illustrate the impact of the combined administration of fentanyl and midazolam on the prognosis of septic patients undergoing invasive mechanical ventilation in the ICU, with incidence-free survival maintained within the groups. **(A)** ICU 28-day mortality; **(B)** hospital 28-day mortality; **(C)** 1-year all-cause mortality.

The results presented in Table 1 indicate that the combination of fentanyl and midazolam is associated with superior survival outcomes, characterized by the lowest LOS in both the ICU and the hospital, as well as reduced ICU and hospital mortality rates. Additionally, this combination was linked to improved 1-year mortality outcomes. To further investigate the impact of fentanyl combined with midazolam, we conducted Cox regression analyses and Kaplan-Meier survival curve assessments. Besides, the analyses demonstrated that the administration of midazolam plus fentanyl was significantly correlated with a shorter ICU and hospital LOS, as well as enhanced 1-year survival rates (Table 2). In the fully adjusted model (Model 3), patients treated with midazolam plus fentanyl showed a higher sHR of ICU discharge (sHR 1.37, 95% CI 1.19–1.57, *P* < 0.001) and hospital discharge (sHR 1.89, 95% CI 1.63–2.19, *P* < 0.001) compared with those receiving other sedation protocols, indicating a shorter ICU and hospital LOS in the presence of the competing risk of death. Moreover, this cohort demonstrated a significantly increased probability of 1-year survival (HR 0.62, 95% CI 0.52–0.74, *P* < 0.001). These associations persisted across both crude and progressively adjusted models, underscoring the robust relationship between the fentanyl plus midazolam regimen and enhanced patient outcomes. Subsequent Kaplan-Meier analysis revealed significant differences in 30-day ICU survival (*P* < 0.001, Figure 2A), 30-day hospital survival (*P* < 0.001, Figure 2B), and 1-year mortality among different sedation regimens (*P* < 0.001, Figure 2C). Patients administered fentanyl in combination with midazolam exhibited the highest survival probability. Furthermore, after performing multivariable logistic regression to evaluate the association between concomitant midazolam and fentanyl exposure and outcomes, we found that midazolam plus fentanyl use was independently associated with lower ICU mortality (OR 0.62, 95% CI 0.46‐0.85, P < 0.001) and lower in‐hospital mortality (OR 0.45, 95% CI 0.33–0.60, P < 0.001) after adjustment for a range of potential confounders (Table 3).

**TABLE 2:** Association of midazolam plus fentanyl with ICU and hospital length of stay and 1-year survival.

Categories	Exposure	Crude model		Model 1		Model 2		Model 3	
		sHR (95%CI)	P value	sHR (95%CI)	P value	sHR (95%CI)	P value	sHR (95%CI)	P value
LOS in ICU	Others	Ref.		Ref.		Ref.		Ref.	
	Midazolam plus fentanyl	1.24 (1.09∼1.42)	<0.001	1.24 (1.08∼1.41)	0.002	1.34 (1.16∼1.54)	<0.001	1.37 (1.19∼1.57)	<0.001
LOS in hospital	Others	Ref.		Ref.		Ref.		Ref.	
	Midazolam plus fentanyl	1.53 (1.33∼1.76)	<0.001	1.53 (1.33∼1.76)	<0.001	1.81 (1.56∼2.09)	<0.001	1.89 (1.63∼2.19)	<0.001

Categories	Exposure	Crude model		Model 1		Model 2		Model 3	
		HR (95%CI)	P value	HR (95%CI)	P value	HR (95%CI)	P value	HR (95%CI)	P value
Survival within 1 year	Others	Ref.			Ref.	Ref.	Ref.		
	Midazolam plus fentanyl	0.67 (0.56∼0.79)	<0.001	0.66 (0.56∼0.78)	<0.001	0.64 (0.54∼0.76)	<0.001	0.62 (0.52∼0.74)	<0.001

ICU, and hospital length of stay were analyzed using Fine–Gray competing risk regression, with death treated as a competing event. One-year survival was analyzed using Cox proportional hazards models. Hazard ratios (HRs) and 95% confidence intervals (CIs) were estimated using Cox proportional hazards models.

Crude model: Unadjusted.

Model 1: Adjusted for age, gender, and BMI.

Model 2: Adjusted for age, gender, BMI, comorbidities, shock, ventilation duration, and severity scores including SOFA, SIRS, SAPS II, OASIS, and GCS.

Model 3: Adjusted for all covariates in Model 2, plus medications (antihypertensives, glucocorticoids, nephrotoxic drugs, immunosuppressants) and laboratory variables including WBC, RBC, platelet count, hemoglobin, albumin, sodium, potassium, total calcium, glucose, PTT, INR, ALT, AST, and NT-proBNP.

**TABLE 3:** Logistic regression analysis of the association between midazolam plus fentanyl and ICU mortality and hospital mortality.

Categories	Exposure	Crude model		Model 1		Model 2		Model3	
		Or (95%CI)	*P* value	Or (95%CI)	*P* value	Or (95%CI)	*P* value	Or (95%CI)	*P* value
ICU mortality	Others	Ref.		Ref.		Ref.		Ref.	
	Midazolam plus fentanyl	0.82 (0.63∼1.08)	0.17	0.79 (0.60∼1.04)	0.1	0.68 (0.50∼0.90)	<0.001	0.62 (0.46∼0.85)	<0.001
Hospital mortality	Others	Ref.		Ref.		Ref.		Ref.	
	Midazolam plus fentanyl	0.63 (0.49∼0.81)	<0.001	0.597 (0.46∼0.77)	<0.001	0.49 (0.37∼0.65)	<0.001	0.45 (0.33∼0.60)	<0.001

Odds ratios (ORs) and 95% confidence intervals (CIs) were estimated using logistic regression models.

Crude model: Unadjusted.

Model 1: Adjusted for age, gender, and BMI.

Model 2: Adjusted for age, gender, BMI, comorbidities, shock, ventilation duration, and severity scores including SOFA, APS III, SIRS, SAPS II, OASIS, and GCS.

Model 3: Adjusted for all covariates in Model 2, plus medications (antihypertensives, glucocorticoids, nephrotoxic drugs, immunosuppressants) and laboratory variables including WBC, RBC, platelet count, hemoglobin, albumin, sodium, potassium, total calcium, glucose, PTT, INR, ALT, AST, and NT-proBNP.

### LASSO regression identifies key factors affecting patient survival outcomes

**FIGURE 3:**
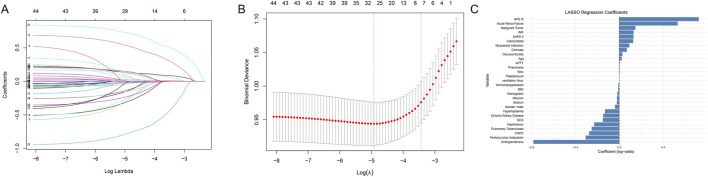
Variable Selection and Risk Estimation Using LASSO Regression. The least absolute shrinkage and selection operator (LASSO) regression model was employed to identify variable selection and coefficient estimation influencing patient mortality in the ICU. **(A)** LASSO regression coefficients. **(B)** Cross-validation plot for the penalty term. **(C)** LASSO-selected variables and their corresponding coefficients.

To further identify key factors influencing patient survival outcomes, we performed a LASSO regression analysis that included fentanyl plus midazolam and other 48 variables in the analysis, specifically age, sex, hypertension, type 2 diabetes, hyperlipidemia, stroke, type 1 diabetes, chronic obstructive pulmonary disease, heart failure, myocardial infarction, malignant tumor, chronic kidney disease, ARF, cirrhosis, hepatitis, tuberculosis, pneumonia, shock, ventilation duration (hours), binary body mass index, SOFA score, APS III score, SIRS score, SAPS II score, OASIS score, GCS level, anti-hypertensive drugs, glucocorticoid, nephrotoxic drug, immunosuppressant, white blood cell count, red blood cell count, platelet count, hemoglobin, albumin, serum sodium, serum potassium, total calcium, blood glucose, partial thromboplastin time, international normalized ratio, alanine aminotransferase, aspartate aminotransferase, and NT-proBNP. As shown in Figures 3A,B, the LASSO regression analysis identified a minimum lambda coefficient of 7. Based on the impact coefficients, the final inclusion criteria comprised fentanyl plus midazolam, age, APS III score, antihypertensive drug, ARF, COPD, and tuberculosis (Figure 3C).

### Subgroup analysis

To further elucidate the impact of the combined administration of fentanyl and midazolam on the prognosis of sepsis patients undergoing mechanical ventilation in the ICU, we conducted subgroup analyses based on key factors identified through LASSO regression, as well as sex.

Cox regression analyses were employed to assess the influence of this sedation and analgesia regimen on patient survival over a 1-year period (Table 4). The subgroup analyses revealed that, after adjusting for other variables, the combination of midazolam and fentanyl was significantly associated with improved 1-year survival outcomes in women (HR 0.51, 95% CI 0.39–0.65), individuals aged 60 years and older (HR 0.58, 95% CI 0.48–0.71), and patients receiving antihypertensive medication (HR 0.56, 95% CI 0.46–0.68), and those with APS III scores ranging from 30 to 60 (HR 0.41, 95% CI 0.31–0.56). Notably, significant differences were observed between sexes, APS III scores (*P* < 0.001), and ARF status (*P* = 0.02). No significant interactions were detected concerning age, use of antihypertensive drugs, COPD, or tuberculosis status.

**TABLE 4:** Subgroup analysis of the association between fentanyl plus midazolam and 1-year survival based on key factors identified by LASSO.

Subgroup	HR (95% CI)	*P* value	*P* for interaction
Gender			
Male	0.75 (0.60, 0.95)	0.02	0.02
Female	0.51 (0.39, 0.65)	<0.001	​
Age, year			
<60	0.77 (0.55, 1.07)	0.12	0.16
≥60	0.58 (0.48, 0.71)	<0.001	​
Antihypertensive			
No	0.88 (0.63, 1.23)	0.45	0.15
Yes	0.56 (0.46, 0.68)	<0.001	​
APS III score	​	​	<0.001
less than 30	0.40 (0.41, 3.90)	0.43	​
(30, 60]	0.41 (0.31, 0.56)	<0.001	​
more than 60	0.81 (0.66, 1.01)	0.07	​
ARF	​	​	0.02
No	0.49 (0.37, 0.66)	<0.001	​
Yes	0.72 (0.58, 0.89)	0.003	​
COPD			
No	0.60 (0.51, 0.72)	<0.001	0.17
Yes	0.99 (0.47, 2.09)	0.98	​
TB	​	​	0.98
No	0.62 (0.52, 0.74)	<0.001	​
Yes	0.58 (0.29, 1.16)	0.12	​

All results were adjusted for the remaining variables.

In addition, a logistic regression analysis was conducted to investigate the impact of the sedation and analgesia regimen on ICU mortality (Table 5). Subgroup analyses revealed that, after adjusting for other variables, the combination of fentanyl and midazolam was associated with a decreased risk of ICU mortality among women (OR 0.89, 95% CI 0.83–0.95), individuals aged 60 years and older (OR 0.89, 95% CI 0.84–0.95), patients receiving antihypertensive medication (OR 0.91, 95% CI 0.87–0.95), and those with APS III scores ranging from 30 to 60 (OR 0.90, 95% CI 0.85–0.95). Significant differences were observed between subgroups based on sex, age, antihypertensive medication use, and APS III score. Conversely, no significant interactions were found for ARF, COPD, and tuberculosis.

**TABLE 5:** Subgroup analysis of the association between fentanyl plus midazolam and ICU mortality based on key factors identified by LASSO.

Subgroup	Or (95% CI)	*P* value	*P* for interaction
Gender			
Male	0.98 (0.92, 1.04)	0.44	0.02
Female	0.89 (0.83, 0.95)	<0.001	​
Age, year			
<60	1.00 (0.95, 1.07)	0.86	0.03
≥60	0.89 (0.84, 0.95)	<0.001	​
Antihypertensive	​	​	0.004
No	1.03 (0.93, 1.13)	0.6	​
Yes	0.91 (0.87, 0.95)	<0.001	​
APS III score	​	​	0.003
less than 30	0.93 (0.82, 1.05)	0.25	​
(30, 60]	0.90 (0.85, 0.95)	<0.001	​
more than 60	0.98 (0.91, 1.05)	0.51	​
ARF	​	​	0.39
No	0.94 (0.89, 0.99)	0.02	​
Yes	0.93 (0.87, 0.99)	0.03	​
COPD			
No	0.93 (0.89, 0.97)	0.002	0.88
Yes	0.97 (0.81, 1.17)	0.77	​
TB	​	​	0.34
No	0.94 (0.89, 0.98)	0.004	​
Yes	0.89 (0.76, 1.03)	0.12	​

All results were adjusted for remaining variables.

### Predictive performance of ML models for ICU mortality and model’s explainability

**FIGURE 4:**
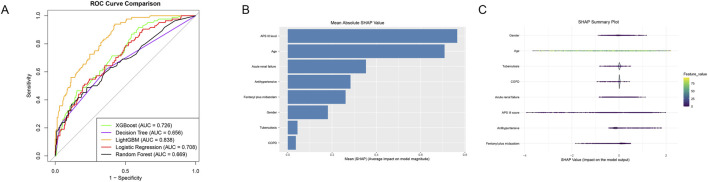
Machine Learning Models and SHAP Interpretation for ICU Mortality Prediction **(A)** The area under the receiver operating characteristic (ROC) curve (AUC) associated with the target diagnosis serves as a predictor of ICU mortality in septic patients receiving mechanical ventilation via tracheal intubation. **(B)** SHAP summary bar plot. **(C)** SHAP summary dot plot.

To optimize the predictive model for survival metrics in septic patients undergoing mechanical ventilation, we developed ICU mortality prediction models utilizing the combined administration of fentanyl and midazolam, alongside key predictors identified through LASSO regression, as well as sex. A comparative analysis of five machine learning algorithms revealed that the LightGBM model exhibited the highest discriminative capability, with an AUC of 0.838, 95%CI (0.795∼0.881). This was followed by the XGBoost model (AUC = 0.726, 95%CI [0.668∼0.784]) and logistic regression (AUC = 0.708, 95%CI [0.647∼0.769]), while the RF (AUC = 0.669, 95%CI [0.601∼0.737]) and DT (AUC = 0.656, 95%CI [0.592∼0.720]) models demonstrated comparatively lower performance (Figure 4A). These results indicate that the LightGBM model may provide superior predictive accuracy for ICU mortality in this patient cohort. To enhance model interpretability and elucidate the contribution of individual variables to mortality risk estimation, we employed the SHAP (SHapley Additive exPlanations) framework. As depicted in Figures 4B,C, SHAP summary plots were generated to illustrate the relative importance of variables within the LightGBM model for septic patients who underwent invasive mechanical ventilation following tracheal intubation. In these plots, features are ranked on the vertical axis based on their overall impact across the population, while the horizontal axis represents the SHAP values, indicating the magnitude and direction of a variable’s effect on the prediction. Each dot corresponds to a specific patient, with its position reflecting the extent to which a particular feature contributed to that individual’s predicted outcome. The analysis identified the most influential predictors for 28-day mortality, ranked in order of decreasing importance, as follows: APS III score, patient age, presence of ARF, administration of antihypertensive medications, use of the fentanyl-midazolam combination, sex, history of tuberculosis, and COPD.

## Discussion

In this study, data sourced from the MIMIC database were systematically analyzed to ascertain the optimal sedation-analgesia regimen for septic patients necessitating endotracheal intubation and invasive mechanical ventilation. Initially, fourteen prevalent sedative and analgesic regimens were assessed utilizing Kaplan–Meier survival analysis alongside Cox proportional hazards modeling. Preliminary findings indicated that the combination of fentanyl and midazolam emerged as the most advantageous regimen concerning both short- and long-term survival outcomes. To enhance the precision of the analysis, critical prognostic factors impacting outcomes within this patient subgroup were identified through LASSO regression. Subsequently, five machine learning algorithms—namely, XGBoost, DT, LightGBM, Logistic Regression, and RF—were employed to construct predictive models. Among these, the LightGBM model exhibited superior performance. SHAP analysis of the LightGBM model elucidated that the most significant predictors of 28-day mortality were the APS III score, patient age, and the presence of acute renal failure.

The selection of an appropriate sedation-analgesia regimen for critically ill patients remains a subject of ongoing debate, particularly for those requiring endotracheal intubation and invasive mechanical ventilation. The significant physiological stress induced by intubation and mechanical ventilation can markedly enhance the patient’s stress response. While deep sedation may potentially attenuate this response, it is also associated with adverse effects, including respiratory and cardiovascular suppression, prolonged mechanical ventilation duration, and an elevated risk of myocardial ischemia in patients with pre-existing cardiac conditions. The latest Society of Critical Care Medicine (SCCM) 2025 guidelines on sedation management in adult ICU patients (Lewis et al., 2025) highlight that most critically ill patients are at high risk for complications such as pain, agitation, and delirium, which have been demonstrated to significantly increase mortality. Numerous studies have indicated that dexmedetomidine may decrease 28-day mortality and the incidence of delirium in patients with sepsis (Aso et al., 2021), while also potentially enhancing sleep quality in critically ill individuals. Nonetheless, evidence remains inconsistent. Certain studies have found that dexmedetomidine does not offer a survival advantage in mechanically ventilated patients, nor does it significantly reduce the duration of ventilation or expedite weaning (Kawazoe et al., 2017). Moreover, recent randomized controlled trials have not demonstrated any significant superiority of dexmedetomidine over conventional sedatives such as propofol or midazolam in terms of reducing neuroinflammatory biomarkers (e.g., S100-β) or improving other markers of sepsis-associated encephalopathy (Iten et al., 2025). Additionally, dexmedetomidine has not been shown to significantly mitigate early stress responses in patients undergoing invasive mechanical ventilation in the ICU setting (Moore et al., 2022). Furthermore, the 2025 SCCM guidelines highlight age-related variability in the efficacy of dexmedetomidine, noting differing sedation responses in patients above and below the age of 65. Our findings indicate that dexmedetomidine, whether administered alone or in conjunction with other sedatives or analgesics, may not constitute the most effective sedation strategy for septic patients undergoing endotracheal intubation. The combination of fentanyl and midazolam yielded favorable outcomes in this patient cohort and there were differences between ages. Fentanyl, a potent opioid analgesic, effectively mitigates the pain response and reduces nociceptive stress, while midazolam, a benzodiazepine sedative, facilitates anxiolysis and decreases metabolic demand. The synergistic application of these agents may contribute to hemodynamic stabilization and attenuation of the stress response, which is particularly advantageous in the context of sepsis. In contrast, propofol—a widely used sedative—has been associated with significant hypotension, potentially exacerbating hemodynamic instability in septic patients.

The APS III is a standardized scoring system extensively utilized in ICUs to evaluate disease severity and predict mortality risk. As an integral component of the broader APACHE II system, it aids clinicians in identifying high-risk patients and optimizing clinical decision-making processes (LeGall et al., 1986). In a comparative study conducted by Fan et al., APS III exhibited superior predictive accuracy for in-hospital mortality among patients with sepsis-associated acute respiratory failure, surpassing other commonly employed scoring systems such as LODS, SAPS II, OASIS, and SOFA (Fan and Ma, 2024). Building upon the identification of the optimal sedation-analgesia regimen in our study, we utilized LASSO regression to ascertain the most critical prognostic factors associated with outcomes in septic patients requiring invasive mechanical ventilation following endotracheal intubation. APS III, age, acute renal failure, and COPD emerged as significant predictors. It is noteworthy that the APS III score inherently incorporates variables related to age and chronic health status. Incorporating age and COPD as distinct variables in our LASSO regression model likely offered a more nuanced depiction of patient risk profiles, thereby complementing the aggregated nature of the APS III score. This approach enhanced the subsequent machine learning models by facilitating more granular and precise predictions of patient outcomes.

Machine learning has fundamentally transformed medical research, particularly within ICU settings, by facilitating dynamic risk stratification and outcome prediction through the analysis of high-dimensional data (Thorsen-Meyer et al., 2020; Shen et al., 2024). In this study, a comparative analysis of five algorithms revealed that LightGBM exhibited superior discriminative capabilities, surpassing XGBoost, logistic regression, and simpler tree-based models. This aligns with its proven efficacy in managing heterogeneous clinical data (Yuan et al., 2025). To mitigate the “black-box” limitation, SHAP analysis identified the APS III score, age, and acute renal failure as the primary predictors of mortality, offering clinically interpretable insights into the contributions of various features. These findings highlight LightGBM’s potential for predicting ICU mortality while maintaining interpretability—a crucial balance for clinical implementation.

This study has several limitations. Firstly, the retrospective nature of the MIMIC database restricts access to consistently recorded real-time assessments of sedation depth, such as RASS scores, and detailed drug titration data. This limitation may introduce residual confounding and constrain the ability to draw causal inferences. Secondly, the reliance on single-center data may hinder the generalizability of the model due to specific patient population characteristics or treatment biases, necessitating further validation to confirm the robustness of the findings. Furthermore, the lack of data on dosage and duration may fail to account for the pharmacokinetic variability of sedatives and analgesics, such as the accumulation of fentanyl in patients with renal impairment. In addition, sedation-related adverse effects and drug withdrawal phenomena were not systematically captured in the database and therefore could not be evaluated across different sedation regimens. Future prospective studies should incorporate dynamic sedation monitoring, drug exposure–response data, and therapeutic drug monitoring to optimize dosing strategies. Additionally, validating the LightGBM model across diverse ICU populations, preferably with temporal or external validation, and integrating it with electronic health record systems could enhance its clinical applicability.

## Conclusion

The results of our study indicate that the combination of fentanyl and midazolam constitutes an effective sedation-analgesia approach for septic patients undergoing endotracheal intubation and invasive mechanical ventilation in the ICU for more than 24 h, leading to enhanced clinical outcomes. Additionally, among the machine learning models devised for mortality prediction, the LightGBM algorithm exhibited the highest performance. Notably, the incorporation of APS III, patient age, and acute renal function yielded a comprehensive predictive model for assessing ICU mortality risk in this critically ill cohort.

## Data Availability

The original contributions presented in the study are included in the article/Supplementary Material, further inquiries can be directed to the corresponding author.
