# Supplementary material for: Machine learning-driven sedation-analgesia optimization in mechanically ventilated sepsis patients: a retrospective MIMIC-IV analysis
Source: Front Pharmacol. 2026 Jan 21;17:1673704. doi: 10.3389/fphar.2026.1673704 (PMC12868193; doi:10.3389/fphar.2026.1673704)
Supplement: Supplementary file 1 [file Table1.pdf]

Variables	Total (n = 2004)	Propofol (n = 8)	Etomidate (n = 1)	Fentanyl(n = 62)	Dexmedetomidine (n = 3)	Midazolam (n = 9)	Hydromorphone (n = 10)	Morphine (n = 28)	Propofol plus fentanyl/hydromorph/morphine (n = 490)	Etomidate plus fentanyl/hydromorph/morphine (n = 158)	Dexmedetomidine plus other drugs (n = 269)	Fentanyl plus midazolam (n = 416)	fentanyl plus midazolam plus hydromorph(n = 123)	Fentanyl plus midazolam plus morphine(n = 236)	Others (n = 191)	*P*
**Demographics**																
Age	64.69 ± 16.72	72.62 ± 10.65	84.00 ± NA	67.19 ± 16.21	66.67 ± 12.01	70.33 ± 18.15	68.10 ± 9.69	70.39 ± 15.94	63.58 ± 17.07	66.27 ± 17.39	60.05 ± 16.83	65.59 ± 16.69	63.05 ± 15.78	69.59 ± 14.02	63.20 ± 17.51	<.001
Gender, n(%)																0.411*
Female	907 (45.26)	5 (62.50)	67 (42.41)	112 (41.64)	202 (48.56)	58 (47.15)	114 (48.31)	91 (47.64)	1 (100.00)	27 (43.55)	1 (33.33)	5 (55.56)	7 (70.00)	11 (39.29)	206 (42.04)	
Male	1097 (54.74)	3 (37.50)	91 (57.59)	157 (58.36)	214 (51.44)	65 (52.85)	122 (51.69)	100 (52.36)	0 (0.00)	35 (56.45)	2 (66.67)	4 (44.44)	3 (30.00)	17 (60.71)	284 (57.96)	
**Marital Status, n(%)**																0.019*
DIVORCED	121 (6.04)	0 (0.00)	3 (1.90)	19 (7.06)	20 (4.81)	7 (5.69)	16 (6.78)	14 (7.33)	0 (0.00)	2 (3.23)	0 (0.00)	0 (0.00)	2 (20.00)	2 (7.14)	36 (7.35)	
MARRIED	864 (43.11)	3 (37.50)	66 (41.77)	94 (34.94)	185 (44.47)	54 (43.90)	117 (49.58)	74 (38.74)	0 (0.00)	27 (43.55)	2 (66.67)	5 (55.56)	5 (50.00)	9 (32.14)	223 (45.51)	
SINGLE	622 (31.04)	3 (37.50)	55 (34.81)	107 (39.78)	131 (31.49)	38 (30.89)	59 (25.00)	60 (31.41)	1 (100.00)	18 (29.03)	0 (0.00)	2 (22.22)	1 (10.00)	10 (35.71)	137 (27.96)	
WIDOWED	234 (11.68)	1 (12.50)	20 (12.66)	18 (6.69)	59 (14.18)	13 (10.57)	31 (13.14)	24 (12.57)	0 (0.00)	8 (12.90)	1 (33.33)	2 (22.22)	1 (10.00)	5 (17.86)	51 (10.41)	
Missing data	163 (8.13)	1 (12.50)	14 (8.86)	31 (11.52)	21 (5.05)	11 (8.94)	13 (5.51)	19 (9.95)	0 (0.00)	7 (11.29)	0 (0.00)	0 (0.00)	1 (10.00)	2 (7.14)	43 (8.78)	
BMI, Mean ± SD	29.08 ± 8.03	27.19 ± 6.35	17.57 ± NA	27.56 ± 6.38	27.31 ± 5.65	28.94 ± 6.82	30.89 ± 8.53	29.41 ± 7.37	28.85 ± 7.52	27.95 ± 7.20	30.66 ± 9.25	29.22 ± 8.62	30.64 ± 8.71	28.13 ± 7.79	28.72 ± 6.84	0.009
BMI binary, n(%)																0.012*
<18.5 or >=25	584 (29.30)	4 (50.00)	47 (30.13)	69 (25.65)	117 (28.47)	24 (19.67)	83 (35.47)	55 (28.80)	0 (0.00)	26 (41.94)	1 (33.33)	1 (11.11)	0 (0.00)	7 (25.00)	150 (30.67)	
18.5-25	1409 (70.70)	4 (50.00)	109 (69.87)	200 (74.35)	294 (71.53)	98 (80.33)	151 (64.53)	136 (71.20)	1 (100.00)	36 (58.06)	2 (66.67)	8 (88.89)	10 (100.00)	21 (75.00)	339 (69.33)	
**Hospitalization and Mortality Outcomes**																
LOS in ICU, days	18.02 ± 14.10	12.17 ± 6.28	9.55 ± NA	14.20 ± 9.29	12.89 ± 5.32	11.16 ± 6.97	16.32 ± 8.21	9.84 ± 7.68	19.47 ± 13.38	22.41 ± 18.59	19.40 ± 12.80	14.25 ± 10.99	21.88 ± 13.99	16.45 ± 16.20	19.58 ± 16.51	<.001
LOS in hosptial, days	10.75 ± 8.98	4.03 ± 2.88	1.31 ± NA	8.71 ± 6.46	6.94 ± 3.16	4.71 ± 2.41	9.11 ± 5.16	6.33 ± 3.56	11.28 ± 8.26	13.19 ± 12.18	12.20 ± 8.93	8.72 ± 7.25	13.30 ± 11.66	10.44 ± 8.50	10.51 ± 10.01	<.001
ICU mortality, n(%)	445 (22.21)	0 (0.00)	38 (24.05)	29 (10.78)	83 (19.95)	15 (12.20)	124 (52.54)	45 (23.56)	0 (0.00)	7 (11.29)	0 (0.00)	2 (22.22)	0 (0.00)	13 (46.43)	89 (18.16)	
Hospital mortality, n(%)	568 (28.34)	0 (0.00)	53 (33.54)	35 (13.01)	91 (21.88)	20 (16.26)	151 (63.98)	67 (35.08)	0 (0.00)	7 (11.29)	0 (0.00)	2 (22.22)	1 (10.00)	16 (57.14)	125 (25.51)	
1-year morlity, n(%)	887 (44.26)	1 (12.50)	81 (51.27)	60 (22.30)	173 (41.59)	40 (32.52)	185 (78.39)	96 (50.26)	1 (100.00)	24 (38.71)	1 (33.33)	3 (33.33)	3 (30.00)	19 (67.86)	200 (40.82)	
Vital sign																
Heart rate, bpm	95.25 ± 21.94	104.75 ± 15.10	99.00 ± NA	91.29 ± 19.61	85.00 ± 14.73	93.67 ± 22.91	81.00 ± 16.89	86.96 ± 17.14	94.26 ± 21.56	97.13 ± 21.63	96.25 ± 22.42	94.70 ± 22.73	97.76 ± 20.94	96.36 ± 21.85	96.14 ± 22.93	0.158
SBP, mmHg	122.64 ± 27.08	137.12 ± 21.81	116.00 ± NA	133.70 ± 29.46	130.00 ± 35.03	132.67 ± 32.24	129.20 ± 25.07	130.68 ± 34.55	123.73 ± 26.62	120.94 ± 25.91	122.87 ± 26.36	120.02 ± 25.19	121.80 ± 28.42	119.50 ± 27.71	124.74 ± 29.34	0.013
DBP, mmHg	67.98 ± 19.48	76.88 ± 30.10	69.00 ± NA	69.84 ± 17.10	63.33 ± 8.50	76.33 ± 22.80	73.80 ± 16.06	66.04 ± 20.86	67.98 ± 18.99	66.96 ± 19.18	69.80 ± 20.73	66.58 ± 17.69	68.47 ± 21.79	67.16 ± 20.13	68.74 ± 20.63	0.618
Respiration rate, bpm	20.92 ± 7.03	21.75 ± 9.38	26.00 ± NA	20.06 ± 7.12	19.67 ± 9.29	19.78 ± 5.85	16.50 ± 3.84	16.79 ± 3.30	21.22 ± 7.37	20.54 ± 7.06	20.53 ± 7.31	21.56 ± 6.92	21.20 ± 6.84	21.16 ± 7.10	20.26 ± 6.07	0.037
SPO_2, %	96.32 ± 5.23	98.62 ± 1.51	99.00 ± NA	97.42 ± 3.73	98.67 ± 1.53	97.11 ± 2.37	98.10 ± 2.18	98.54 ± 2.40	96.22 ± 4.97	96.14 ± 4.92	96.39 ± 7.17	95.83 ± 5.50	96.10 ± 4.56	96.13 ± 4.84	97.08 ± 3.95	0.091
Temperature, ℃	36.69 ± 2.30	37.59 ± 0.62	36.72 ± NA	36.80 ± 1.00	36.63 ± 0.81	36.77 ± 0.64	36.85 ± 1.34	36.81 ± 1.35	36.95 ± 0.68	36.65 ± 0.88	36.92 ± 0.97	36.41 ± 3.35	36.23 ± 4.39	36.44 ± 3.31	36.83 ± 0.96	0.021
Ventilation Hour, Mean ± SD	176.59 ± 186.25	57.61 ± 49.21	27.63 ± NA	132.34 ± 138.58	131.66 ± 73.00	54.98 ± 37.15	137.64 ± 134.69	98.78 ± 57.31	176.27 ± 159.97	222.66 ± 264.86	195.35 ± 178.93	148.06 ± 152.42	224.57 ± 248.95	187.47 ± 182.60	170.72 ± 220.15	<.001
Shock, n(%)																0.417*
0	1800 (90.59)	8 (100.00)	142 (89.87)	242 (90.30)	371 (89.83)	113 (93.39)	203 (87.50)	168 (88.42)	1 (100.00)	59 (96.72)	3 (100.00)	9 (100.00)	10 (100.00)	28 (100.00)	443 (91.34)	
1	187 (9.41)	0 (0.00)	16 (10.13)	26 (9.70)	42 (10.17)	8 (6.61)	29 (12.50)	22 (11.58)	0 (0.00)	2 (3.28)	0 (0.00)	0 (0.00)	0 (0.00)	0 (0.00)	42 (8.66)	
**Site of Inflammatory Involvement**																
Respiration, Mean ± SD	0.91 ± 1.17	0.25 ± 0.71	0.00 ± NA	0.69 ± 1.12	0.67 ± 1.15	1.22 ± 1.20	0.80 ± 1.32	0.86 ± 1.08	0.98 ± 1.16	0.84 ± 1.22	1.23 ± 1.25	0.85 ± 1.17	0.79 ± 1.11	0.76 ± 1.12	0.81 ± 1.14	<.001
Coagulation, Mean ± SD	0.46 ± 0.86	0.00 ± 0.00	0.00 ± NA	0.35 ± 0.85	1.00 ± 1.00	0.44 ± 1.01	0.20 ± 0.63	0.39 ± 0.74	0.49 ± 0.88	0.56 ± 0.96	0.39 ± 0.82	0.39 ± 0.80	0.37 ± 0.78	0.58 ± 0.98	0.52 ± 0.82	0.094
Liver, Mean ± SD	0.33 ± 0.88	0.25 ± 0.71	0.00 ± NA	0.29 ± 0.86	1.00 ± 1.73	0.33 ± 1.00	0.00 ± 0.00	0.36 ± 0.99	0.28 ± 0.79	0.40 ± 0.98	0.26 ± 0.75	0.33 ± 0.88	0.29 ± 0.80	0.45 ± 1.02	0.47 ± 0.98	0.187
Cardiovascular, Mean ± SD	1.10 ± 1.26	1.00 ± 1.31	0.00 ± NA	0.84 ± 0.91	0.67 ± 0.58	1.00 ± 1.22	0.90 ± 0.88	0.93 ± 1.27	1.08 ± 1.24	1.04 ± 1.16	1.11 ± 1.27	1.22 ± 1.34	1.00 ± 1.19	1.29 ± 1.38	0.88 ± 1.16	0.080
Cns, Mean ± SD	0.50 ± 0.98	1.00 ± 1.31	3.00 ± NA	0.73 ± 1.13	0.67 ± 1.15	1.67 ± 1.41	1.10 ± 1.45	0.75 ± 1.38	0.48 ± 0.93	0.48 ± 0.91	0.49 ± 1.03	0.45 ± 0.97	0.45 ± 0.85	0.48 ± 0.95	0.54 ± 1.02	0.002
Renal, Mean ± SD	0.60 ± 1.03	0.75 ± 1.16	0.00 ± NA	0.58 ± 0.95	0.67 ± 0.58	1.11 ± 1.45	0.90 ± 1.37	0.46 ± 0.69	0.50 ± 0.94	0.59 ± 0.98	0.49 ± 0.97	0.73 ± 1.14	0.67 ± 1.06	0.69 ± 1.05	0.62 ± 1.05	0.080
**Clinical Severity Scores**																
Sirs, Mean ± SD	3.06 ± 0.85	3.50 ± 0.76	2.00 ± NA	2.82 ± 0.88	2.67 ± 0.58	2.89 ± 0.93	2.80 ± 1.14	3.14 ± 0.85	2.94 ± 0.87	2.97 ± 0.84	3.05 ± 0.86	3.16 ± 0.88	3.15 ± 0.80	3.24 ± 0.77	3.08 ± 0.80	<.001
Sofa, Mean ± SD	7.02 ± 3.98	6.12 ± 3.72	4.00 ± NA	6.19 ± 3.64	6.33 ± 3.06	5.67 ± 3.24	5.30 ± 3.47	5.75 ± 3.60	6.64 ± 4.07	6.73 ± 3.31	6.73 ± 3.91	7.50 ± 3.94	7.07 ± 4.25	8.41 ± 4.07	6.52 ± 3.97	<.001
Apsiii, Mean ± SD	60.16 ± 24.37	59.25 ± 25.98	51.00 ± NA	57.15 ± 20.80	51.33 ± 29.94	61.67 ± 19.02	48.20 ± 19.72	56.61 ± 25.36	56.37 ± 23.44	59.22 ± 21.50	56.59 ± 24.01	64.48 ± 24.77	62.29 ± 25.17	68.23 ± 24.07	57.22 ± 26.16	<.001
Sapsii, Mean ± SD	45.15 ± 15.54	44.88 ± 8.22	52.00 ± NA	43.18 ± 14.45	39.67 ± 11.68	49.89 ± 16.37	39.80 ± 13.39	44.82 ± 13.28	43.33 ± 14.82	43.59 ± 14.78	42.55 ± 14.80	46.89 ± 16.00	45.10 ± 15.04	51.63 ± 15.74	43.77 ± 16.73	<.001
Oasis, Mean ± SD	38.71 ± 8.38	38.88 ± 7.51	45.00 ± NA	37.21 ± 7.83	35.33 ± 9.02	41.22 ± 8.18	37.40 ± 6.13	37.93 ± 7.84	38.10 ± 8.40	37.98 ± 8.87	37.67 ± 8.10	39.83 ± 8.14	38.72 ± 8.16	41.27 ± 8.30	37.27 ± 8.62	<.001
Gcs, Mean ± SD	12.80 ± 3.53	11.62 ± 3.96	7.00 ± NA	11.75 ± 3.85	11.67 ± 2.89	11.33 ± 3.91	12.80 ± 3.08	11.43 ± 4.84	12.94 ± 3.45	13.27 ± 2.99	12.91 ± 3.54	12.92 ± 3.46	13.00 ± 3.36	12.44 ± 3.91	12.60 ± 3.51	0.046
**Laboratory Findings**																
WBC, K/μL	13.27 ± 8.93	10.88 ± 2.02	9.30 ± NA	13.31 ± 11.34	11.07 ± 5.14	14.97 ± 19.09	9.56 ± 3.11	12.64 ± 3.65	13.50 ± 8.87	13.22 ± 7.40	13.53 ± 8.76	12.51 ± 7.73	14.12 ± 8.78	13.53 ± 8.88	13.50 ± 11.98	0.837
RBC, m/μL	3.59 ± 0.78	3.58 ± 0.53	3.91 ± NA	3.63 ± 0.77	3.28 ± 0.36	3.75 ± 0.63	3.55 ± 0.41	3.83 ± 0.81	3.72 ± 0.82	3.44 ± 0.73	3.63 ± 0.80	3.56 ± 0.74	3.57 ± 0.77	3.47 ± 0.74	3.54 ± 0.77	0.003
Plt, K/μL	207.45 ± 114.99	223.50 ± 114.65	246.00 ± NA	216.81 ± 99.32	133.00 ± 88.83	214.22 ± 106.25	241.40 ± 93.60	189.79 ± 82.53	203.57 ± 114.39	204.62 ± 125.69	208.20 ± 110.16	215.07 ± 110.38	222.61 ± 122.83	206.69 ± 130.98	191.14 ± 107.85	0.532
HGB, mg/dL	10.80 ± 2.26	10.31 ± 1.66	13.10 ± NA	10.80 ± 1.86	11.10 ± 1.67	11.33 ± 2.10	10.15 ± 1.35	11.29 ± 2.75	11.15 ± 2.42	10.39 ± 2.21	10.96 ± 2.34	10.71 ± 2.11	10.58 ± 2.28	10.41 ± 2.12	10.75 ± 2.21	0.003
Albumin, g/dL	2.88 ± 0.63	3.37 ± 0.64	3.60 ± NA	2.92 ± 0.65	3.60 ± NA	2.64 ± 0.52	3.43 ± 0.42	3.36 ± 0.67	2.89 ± 0.64	2.88 ± 0.64	2.97 ± 0.58	2.85 ± 0.62	2.68 ± 0.62	2.77 ± 0.61	2.98 ± 0.68	<.001
Sodium, mEq/L	138.39 ± 5.83	136.88 ± 9.25	131.00 ± NA	138.55 ± 6.28	140.33 ± 4.51	140.89 ± 3.82	138.30 ± 5.14	139.36 ± 4.75	138.29 ± 5.45	137.34 ± 5.36	138.77 ± 5.30	138.84 ± 6.54	137.84 ± 5.44	137.76 ± 6.53	138.92 ± 5.35	0.115
Potassium, mEq/L	4.19 ± 0.80	3.80 ± 0.67	3.50 ± NA	4.07 ± 0.93	3.80 ± 0.46	3.94 ± 0.68	4.13 ± 0.73	4.03 ± 0.89	4.15 ± 0.74	4.23 ± 0.75	4.23 ± 0.83	4.21 ± 0.89	4.20 ± 0.77	4.22 ± 0.78	4.18 ± 0.71	0.704
Calcium, mEq/L	8.07 ± 0.96	8.31 ± 0.79	8.50 ± NA	8.21 ± 0.81	8.27 ± 0.31	7.77 ± 1.04	8.24 ± 0.45	8.44 ± 0.60	8.15 ± 0.94	8.06 ± 0.96	8.10 ± 0.99	7.94 ± 0.96	7.90 ± 1.00	8.01 ± 1.00	8.24 ± 0.99	0.007
Glucose, mg/dL	154.55 ± 81.32	154.75 ± 84.54	114.00 ± NA	153.79 ± 64.48	134.00 ± 29.82	171.44 ± 120.54	142.90 ± 44.93	138.21 ± 64.04	152.36 ± 70.00	144.92 ± 58.15	146.08 ± 61.36	164.67 ± 110.14	158.02 ± 101.31	163.64 ± 85.60	147.49 ± 57.45	0.132
PT, sec	17.51 ± 11.36	14.42 ± 3.03	14.00 ± NA	19.17 ± 19.28	13.47 ± 1.29	15.99 ± 6.92	15.56 ± 9.05	18.41 ± 9.67	16.65 ± 10.84	17.49 ± 10.51	16.60 ± 11.03	18.59 ± 12.78	17.13 ± 13.33	18.98 ± 10.05	16.80 ± 7.05	0.277
APTT, sec	40.04 ± 24.16	31.68 ± 4.22	27.60 ± NA	38.01 ± 17.30	27.97 ± 2.27	32.14 ± 12.94	29.28 ± 6.20	40.00 ± 27.14	40.02 ± 24.01	38.44 ± 19.81	39.06 ± 23.91	40.82 ± 25.06	38.16 ± 24.33	43.35 ± 25.90	40.33 ± 26.53	0.578
INR	1.59 ± 1.06	1.35 ± 0.29	1.20 ± NA	1.54 ± 0.91	1.17 ± 0.12	1.43 ± 0.73	1.42 ± 1.01	1.72 ± 1.12	1.52 ± 0.85	1.62 ± 1.35	1.50 ± 1.03	1.71 ± 1.39	1.44 ± 0.56	1.76 ± 1.06	1.53 ± 0.73	0.088
D-Dimer	3956.57 ± 2411.20	NaN ± NA	NaN ± NA	NaN ± NA	NaN ± NA	NaN ± NA	NaN ± NA	NaN ± NA	NaN ± NA	1665.00 ± NA	NaN ± NA	4990.29 ± 3185.13	3432.00 ± NA	3585.18 ± 2019.20	3622.00 ± NA	0.684
NTproBNP	10478.62 ± 13762.14	8589.00 ± NA	NaN ± NA	10214.50 ± 11803	13614.00 ± NA	NaN ± NA	17156.00 ± NA	20213.00 ± 20806.08	8105.10 ± 11065.54	10198.57 ± 10935.32	8196.65 ± 13486.77	9467.19 ± 14170.97	9788.91 ± 13391.39	15490.18 ± 15593.10	18109.17 ± 20230.88	0.459
**ICU Medications**																
Antihypertensive																<.001*
Yes	1618 (80.74)	6 (75.00)	141 (89.24)	238 (88.48)	317 (76.20)	102 (82.93)	178 (75.42)	150 (78.53)	0 (0.00)	45 (72.58)	3 (100.00)	6 (66.67)	9 (90.00)	17 (60.71)	406 (82.86)	
No	386 (19.26)	2 (25.00)	17 (10.76)	31 (11.52)	99 (23.80)	21 (17.07)	58 (24.58)	41 (21.47)	1 (100.00)	17 (27.42)	0 (0.00)	3 (33.33)	1 (10.00)	11 (39.29)	84 (17.14)	
Glucocorticoids																0.024*
Yes	553 (27.59)	3 (37.50)	35 (22.15)	60 (22.30)	139 (33.41)	28 (22.76)	71 (30.08)	56 (29.32)	0 (0.00)	14 (22.58)	0 (0.00)	0 (0.00)	4 (40.00)	5 (17.86)	138 (28.16)	
No	1451 (72.41)	5 (62.50)	123 (77.85)	209 (77.70)	277 (66.59)	95 (77.24)	165 (69.92)	135 (70.68)	1 (100.00)	48 (77.42)	3 (100.00)	9 (100.00)	6 (60.00)	23 (82.14)	352 (71.84)	
Nephrotoxic																0.003*
Yes	1707 (85.18)	7 (87.50)	144 (91.14)	237 (88.10)	352 (84.62)	115 (93.50)	202 (85.59)	154 (80.63)	0 (0.00)	49 (79.03)	2 (66.67)	8 (88.89)	7 (70.00)	22 (78.57)	408 (83.27)	
No	297 (14.82)	1 (12.50)	14 (8.86)	32 (11.90)	64 (15.38)	8 (6.50)	34 (14.41)	37 (19.37)	1 (100.00)	13 (20.97)	1 (33.33)	1 (11.11)	3 (30.00)	6 (21.43)	82 (16.73)	
Immunosuppressant																0.050*
Yes	77 (3.84)	0 (0.00)	6 (3.80)	2 (0.74)	22 (5.29)	4 (3.25)	6 (2.54)	14 (7.33)	0 (0.00)	2 (3.23)	0 (0.00)	1 (11.11)	0 (0.00)	1 (3.57)	19 (3.88)	
No	1927 (96.16)	8 (100.00)	152 (96.20)	267 (99.26)	394 (94.71)	119 (96.75)	230 (97.46)	177 (92.67)	1 (100.00)	60 (96.77)	3 (100.00)	8 (88.89)	10 (100.00)	27 (96.43)	471 (96.12)	
**Comorbidity, n(%)**																0.003*
No	109 (5.44)	0 (0.00)	6 (3.80)	21 (7.81)	16 (3.85)	10 (8.13)	4 (1.69)	23 (12.04)	0 (0.00)	4 (6.45)	0 (0.00)	1 (11.11)	0 (0.00)	1 (3.57)	23 (4.69)	
Yes	1895 (94.56)	8 (100.00)	152 (96.20)	248 (92.19)	400 (96.15)	113 (91.87)	232 (98.31)	168 (87.96)	1 (100.00)	58 (93.55)	3 (100.00)	8 (88.89)	10 (100.00)	27 (96.43)	467 (95.31)	
